# Supplementary material for: High-throughput and automatic structural and developmental root phenotyping on Arabidopsis seedlings
Source: Plant Methods. 2022 Dec 1;18:127. doi: 10.1186/s13007-022-00960-5 (PMC9714072; doi:10.1186/s13007-022-00960-5)
Supplement: Supplementary file 1 — Additional file 1: Figure S1. Successive p-values obtained at each time step with the Mann–Whitney test. This corresponds to the null hypothesis H0:”At each timestep, the mean growth of the root systems under osmotic stress and the mean growth of the control root systems are equal.” (see Fig. 9 for the original data). [file 13007_2022_960_MOESM1_ESM.docx]

**High-throughput and automatic structural and developmental root phenotyping on Arabidopsis seedlings**

**Authors:**

Romain Fernandez^1,2^, Amandine Crabos^3^, Morgan Maillard^3^, Philippe Nacry*^3^, Christophe Pradal*^1,2,4^

^1^CIRAD, UMR AGAP Institut, F-34398 Montpellier, France.

^2^UMR AGAP Institut, Univ Montpellier, CIRAD, INRAE, Institut Agro, F-34398 Montpellier, France.

^3^Institute for Plant Sciences of Montpellier (IPSiM), Univ Montpellier, CNRS, INRAE, Institut Agro, Montpellier, France.

^4^Inria & LIRMM, Univ Montpellier, CNRS, Montpellier, France.

*Co-corresponding authors

**Corresponding authors:**

Philippe Nacry: [philippe.nacry@inrae.fr](mailto:philippe.nacry@inrae.fr)

Christophe Pradal: [christophe.pradal@cirad.fr](mailto:christophe.pradal@cirad.fr)


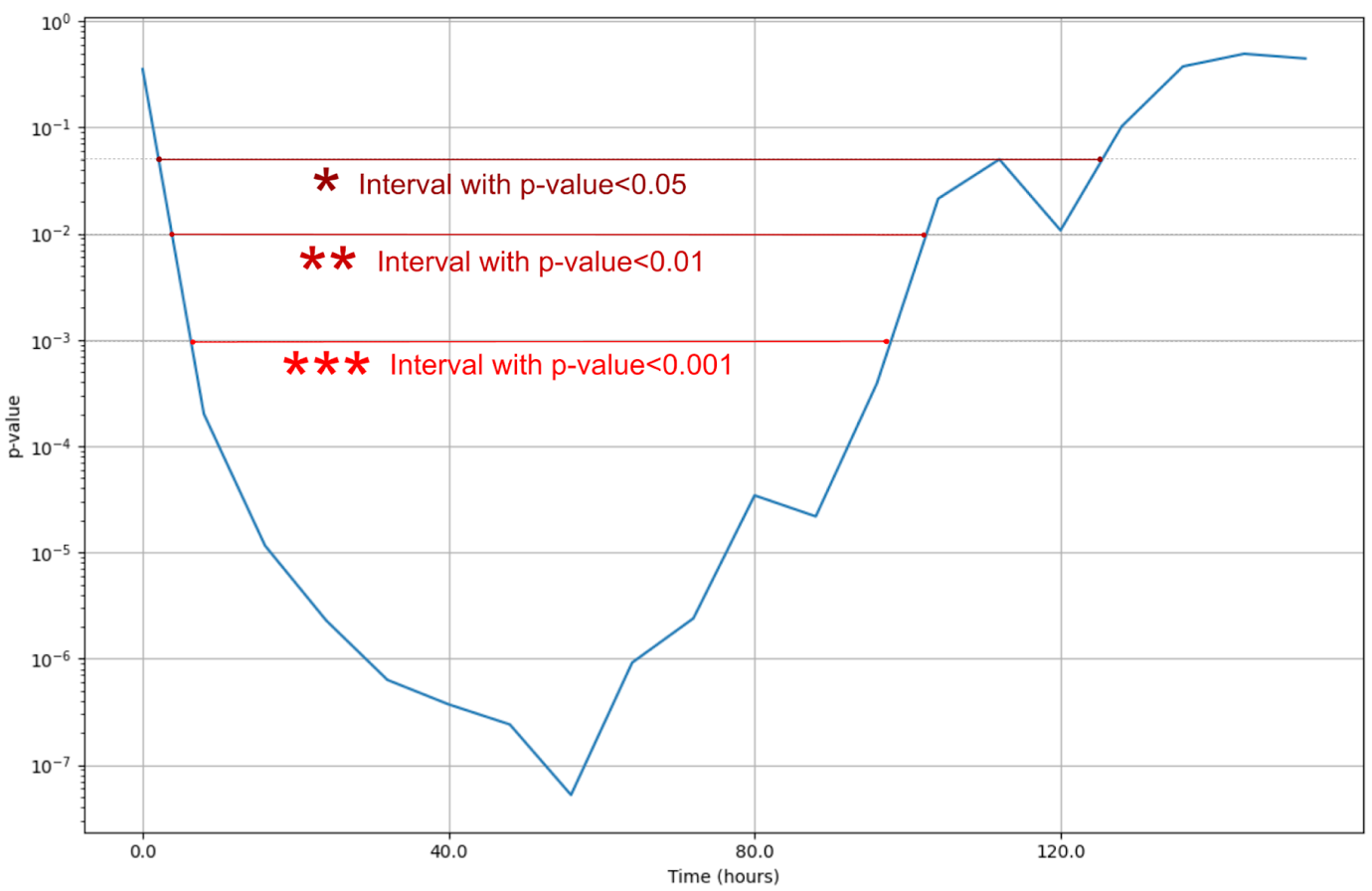


**Figure S1:** *Successive p-values obtained at each time step with the Mann-Whitney test. This corresponds to the null hypothesis H0: ”At each timestep, the mean growth of the root systems under osmotic stress and the mean growth of the control root systems are equal.” (see Figure 9 for the original data).*
